# Identification of bisphenols and derivatives in greenhouse dust as a potential source for human occupational exposure

**DOI:** 10.1007/s00216-021-03863-x

**Published:** 2022-01-29

**Authors:** Noelia Caballero-Casero, Soledad Rubio

**Affiliations:** Department of Analytical Chemistry, Institute of Fine Chemistry and Nanochemistry, Edificio Anexo Marie Curie, Campus de Rabanales, 14071 Córdoba, Spain

**Keywords:** Greenhouse dust, Supramolecular solvent, Liquid chromatography–tandem mass spectrometry, Risk assessment, Bisphenols, Occupational exposure

## Abstract

Bisphenol A (BPA) and alternative bisphenols are widely used in the industrial production of polycarbonates and resin polymers. Adverse effects on human health have been described for BPA and owing to the structural similarity of alternative bisphenols and derivatives, a similar toxicity profile is expectable. Dust can act as a sink for bisphenols owing to the large surface area to mass ratio. Human risk exposure to bisphenols via indoor dust has been widely assessed in the last decade. The environmental conditions inside greenhouses, among other factors, facilitate that chemicals are released from greenhouse building materials to dust. This study aims to explore for the first time the potential of greenhouse dust as a new source of bisphenols for human exposure. For this purpose, a supramolecular solvent-based method was applied to the extraction of twenty-one bisphenols from greenhouse dust, prior to their determination by liquid chromatography–tandem mass spectrometry. Nineteen bisphenols were found in the five greenhouse dust samples analysed, with concentrations ranging from 5275 ng g^−1^ (BPA) to 0.25 ng g^−1^ (trichlorobisphenol A). The average daily dose (ADD) via dust ingestion for bisphenol compounds was calculated, in order to estimate the occupational exposure for inadvertent dust ingestion. Despite the calculated ADD value for BPA (47.81 ng kg^−1^ day^−1^) being below the tolerable daily intake proposed by EFSA (4·10^3^ ng kg^−1^ day^−1^), this value was considerably higher than those previously reported for indoor dust, which brings to light the importance of considering greenhouse dust as bisphenols source of exposure for greenhouse workers.

**Graphical abstract:**

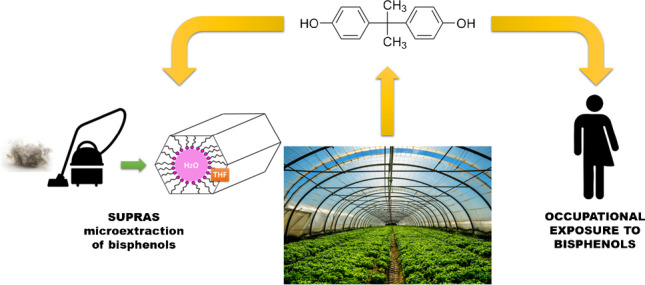

## Introduction

Bisphenol A (BPA) is a synthetic high-production chemical used as a monomer in the production of polycarbonate plastic, epoxy resins and other plastics polymers. BPA is an androgenic and estrogenic disruptor [1], and it has been classified by the European Union (EU) as a reproductive toxicant category 1B [2]. Various toxicological effects on humans have been reported for BPA: adverse effects on the reproductive, immune and cognitive system; obesity, metabolic dysfunctions, diabetes and behavioural development [3]. The raising concern about BPA effects had led to the development of regulatory legislation and the establishment of maximum levels permitted for several applications. As a result, the industry has replaced BPA with other bisphenol compounds in some applications. For example, BPS is widely used as a colour developer in thermal paper [4]. BPA-chlorinated derivatives are formed due to the reaction of BPA with chlorine atoms, for example with the sodium hypochlorite used for disinfection in tap water. Bisphenol diglycidyl ethers are used as building blocks of epoxy resins and other plastic polymers, which can be transformed into hydrolytic and chlorinated forms under humid or acidic conditions [5–7]. Unfortunately, the alternative bisphenols are structurally similar to BPA, so exhibit a similar toxicity profile but being even less well-known than BPA [8, 9].

Indoor dust has been proved to act as a sink for multiple chemical classes owing to the large surface area to mass ratio. Indoor dust from residential, vehicles and workplaces (offices) have been detected as important sources of human exposure to semivolatile organic compounds such as pesticides, (new)flames retardants and plasticizers [7, 10, 11]. Typically, chemicals reach the dust either via direct application into the indoor environment, for example pesticides; or by releasing from materials and consumer products (volatilization of chemicals, transfer for contact material-dust, abrasion of particles from a product) as in the case of bisphenols [12, 13].

Human exposure to dust occurs through two different pathways: (i) via dermal contact and (ii) via dust ingestion [14]. Inhalation exposure to dust is minimum and usually is neglected, albeit under some conditions may be an important exposure route. Dermal exposure depends on the skin surface exposed, the diameter of dust particles and the dust adherence to skin factor [15]. The average rates of dust ingestion are yet uncertain, although is strongly affected by the dust particle diameter and adherence to skin and the hand-to-mouth behaviour frequency [14]. Although dust ingestion might be a minor pathway for bisphenols compared to bisphenol dietary intake, from an occupational point of view, the high concentration and/or exposure time may contribute substantially to the total exposure. Human risk exposure to bisphenols via indoor dust has been widely assessed in the last decade. Dust samples have been collected from resdentials, vehicles, offices, plastic-related industries and schools, among others, over the world [7, 16, 17]. However, there are still potential sources of bisphenol exposure that remain unknown.

The aim of this research focuses on the existing gap regarding the contribution of dust ingestion to the total greenhouse workers’ exposure to bisphenols, chlorinated derivatives and diglycidyl ethers. Industrial greenhouses are made of plastic materials such as polycarbonates and polymers. The environmental conditions inside greenhouses, such as continuous radiation, high temperature and humidity, together with the use of phytosanitary products promote the degradation of greenhouse building materials. Thus, chemicals can release from greenhouse building materials to dust.

The occurrence of 21 bisphenols and derivatives in dust from greenhouses was determined by the combined use of supramolecular solvents (SUPRAS) and liquid chromatography–tandem mass spectrometry (LC–MS/MS). SUPRAS are environmentally responsive nanostructured liquids made up of colloidal suspensions of amphiphiles produced in a process of spontaneous self-assembly and coacervation [18]. The supramolecular aggregate formation is driven by non-covalent interactions; thus, they are reversible and can be tuneable by tailoring the synthesis conditions. SUPRAS have outstanding properties for multicompounds solubilisation, mainly (i) a high number of binding sites; (ii) two microenvironments of different polarity; and (iii) ability to behave as restricted access materials by excluding interferents through physical and chemical mechanisms [18–20]. The combination of these properties makes SUPRAS excellent solvents that can solubilise simultaneously multiple organic compounds by dispersion forces, hydrogen bondings, polar interactions, etc., getting clean extracts. In this way, they can efficiently extract bisphenols and derivatives in a wide polarity range (log *K*_o/w_ from 1.25 to 6.56), as previously proved for their analysis in common human exposure sources to bisphenols [21].

To the best of our knowledge, this is the first proposal to identify the potential risk of occupational exposure to bisphenols in greenhouse dust. The obtained results and main conclusions are exposed and discussed below.

## Materials and methods

### Chemicals and reagents

All chemicals were of analytical grade and were used as supplied. The name and acronyms of the twenty-one bisphenols investigated are specified in Table 1. BPA, BPF, BPP, BPS, BPZ, BPAF, BPAP, BADGE, BADGE·H_2_O, BADGE·2H_2_O, BADGE·HCl, BADGE·2HCl, BADGE·H_2_O·HCl, BFDGE and BFDGE·2H_2_O were supplied by Sigma-Aldrich (Steinheim, Germany). MCBPA, DCBPA, TCBPA and TeCBPA were obtained from Cymit (Barcelona, Spain). BPB and BPE were supplied by TCI Europe (Zwijndrecht, Belgium). Labelled isotopically bisphenol A (^13^C-BPA) and bisphenol A diglycidyl ether (d_6_-BADGE) were acquired from Cambridge isotope laboratories (UK). Methanol, 1-hexanol (H) and tetrahydrofuran (THF) were purchased from VWR-Prolabo (Bois, France). Ammonium formate (≥ 99%) was supplied by Sigma-Aldrich (St. Louis, USA) and formic acid (98%) by Panreac Química (Barcelona, Spain). Ultra-pure quality water was obtained from a milli-Q water purification system (Millipore, Madrid, Spain), and Lichrosolv® water was supplied by Merck KGaA (Darmstadt, Germany).

**Table 1:**
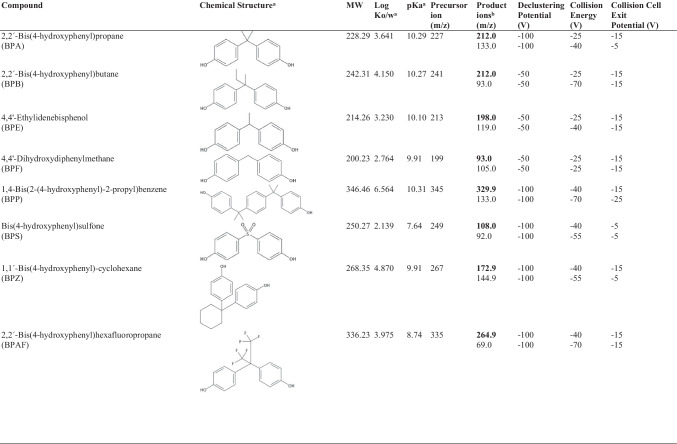

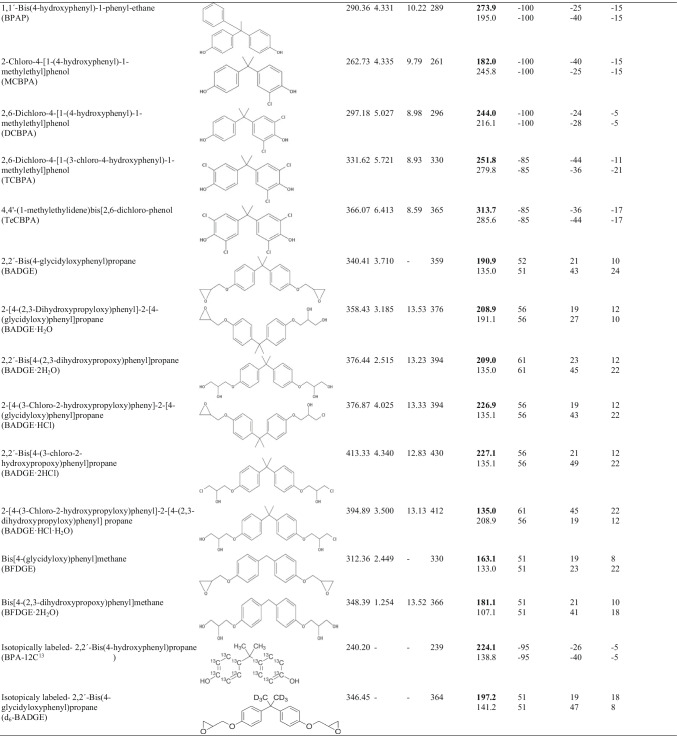
Chemical information and MS parameters used for the quantification of bisphenols and derivatives. ^a^Obtained from Scifinder Scholar. Available from: https://scifinder.cas.org. ^b^Quantifier (in bold) and qualifier ions

Stock solutions were prepared for individual bisphenols or internal standards in methanol at 1–2.5 g L^−1^ range and stored at − 20 °C until their use. Intermediate solutions of bisphenol mixtures were prepared in methanol at a concentration of 10 mg L^−1^. Working solutions were prepared weekly by appropriate dilution of the intermediate solutions with methanol.

### *Sample collection*

**Fig. 1:**
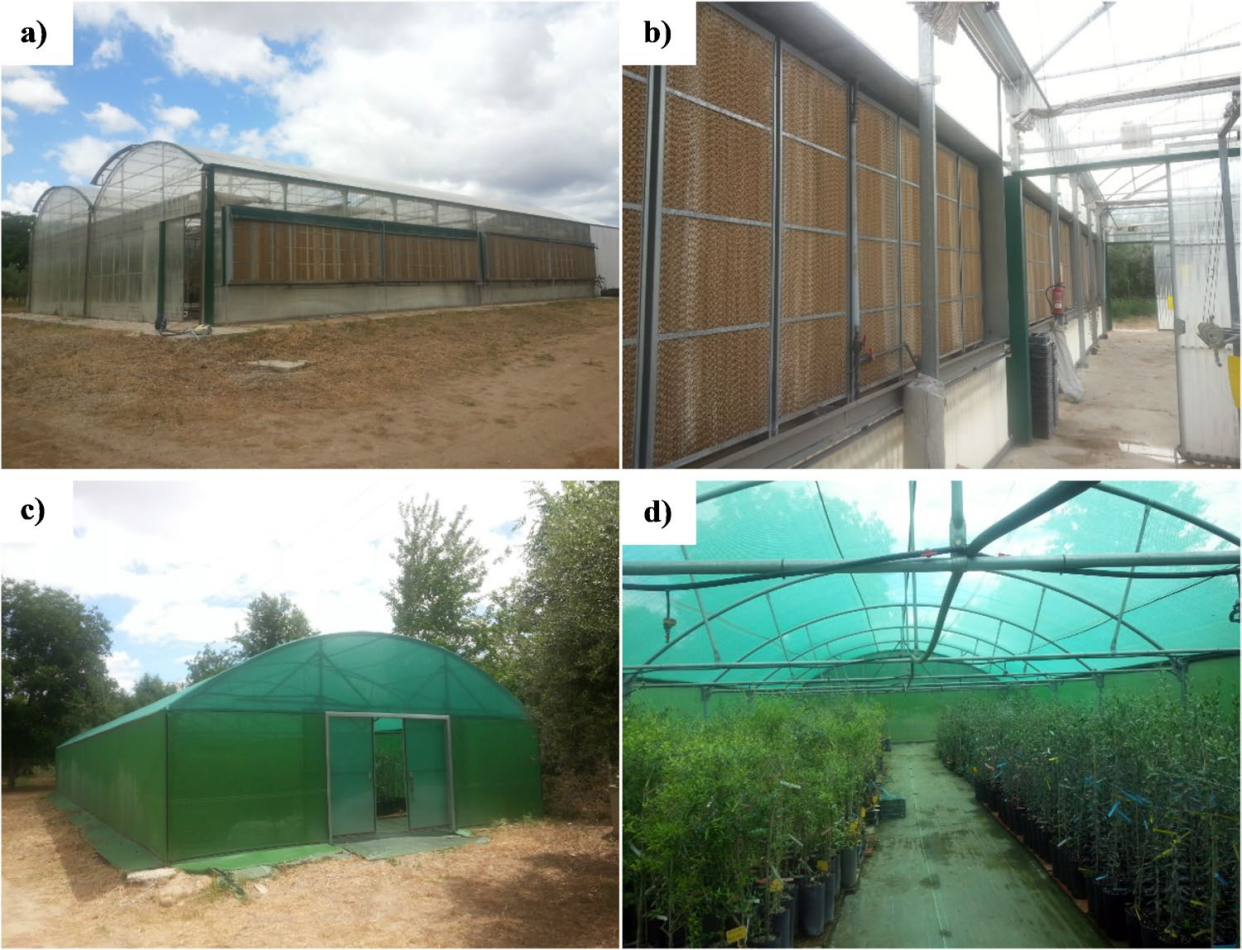
Images of two different greenhouses: a polycarbonate sawtooth greenhouse (**a**) and its evaporative cooling system (**b**); and a polyethylene-geotextile shade house greenhouse (**c**). The photo (**d**) shows the inside of the shade house greenhouse

Dust samples (*n* = 5) were collected in two representative types of greenhouses (Fig. 1) from Andalusia, Spain. The sawtooth greenhouse is made of polycarbonate over a metallic structure (Fig. 1a). Ventilation and humidity levels are controlled with an evaporative cooling system and a set of fans (Fig. 1b). Moreover, 1.8 m over the floor, there are lamps to provide extra radiation because of its positive effect on seedling growth. A regular vacuum cleaner was operated, equipped with a paper deposition bag with an inline fibre filter to hold dust inside, to collect dust from the surface of lamps (sample 1), polycarbonate panels (sample 2), evaporative cooling system (sample 3) and fans (sample 4). The second greenhouse is a shade house type made of polyethylene-based geotextile over a metallic structure (Fig. 1c–d); thus, there is a constant flow of air between the inside and outside; and the humidity level is equivalent to the outside environment. Sample 5 was collected directly from the geotextile walls. Special care was taken in order not to collect ground particles from soil. To avoid cross-contamination, dust was collected in separate bags for each location and the vacuum cleaner was cleaned with methanol between samples. The collection bags were labelled with a pencil to avoid sample contamination from the pen ink.

Fibres, hair and big particles were removed from dust samples with the help of metallic tweezers. Then, dust samples were sieved using a pre-cleaned metallic double sieve of 1000 and 500 µm of pore size. Finally, the samples were individually stored in dark glass bottles at room temperature until their analysis.

### Control of background bisphenol contamination

Bisphenols are ubiquitous compounds; therefore, it is crucial to reduce the potential source of contamination. All the experiments were carried out in a dedicated room where all the surfaces every day were thoroughly cleaned using methanol. Whenever possible, only glass labware was used, which was successively sonicated in distilled water with mild detergent, distilled water and methanol (twice each step), immediately of being used. The unavoidable plastic material (i.e. microtubes and pipettes tips) was rinsed with methanol before their use. Bisphenols can leach from several components of the chromatographic system; thus, symmetry C18 column (3.5 µm, 75 mm × 4.6 mm. Waters (Milford, MA, USA)) was placed between the binary pump and the automatic sampler. Therefore, bisphenols coming from the chromatographic devices elute later on the chromatogram. In addition, ultra-pure quality water was filtered through Empore SDB-XC disks (Análisis Vínicos, Tomelloso, Spain), for removing potential BPA.

### SUPRAS -based microextraction of bisphenol compounds

First, hexanol-based SUPRAS-RAM was synthesized by mixing 1-hexanol (3 mL, 10% v/v/v), THF (6 mL, 20% v/v/v) and water (21 mL, 70% v/v/v) in a 50-mL glass centrifuge tube. Supramolecular aggregates were spontaneously formed and the mixture was centrifuged for 30 min at 3500 rpm to accelerate SUPRAS phase separation from the bulk solution. The obtained SUPRAS volume (6.2 mL), able to treat ~ 15 samples, was collected with a syringe and kept at room temperature in an airtight vial until its use.

**Fig. 2:**
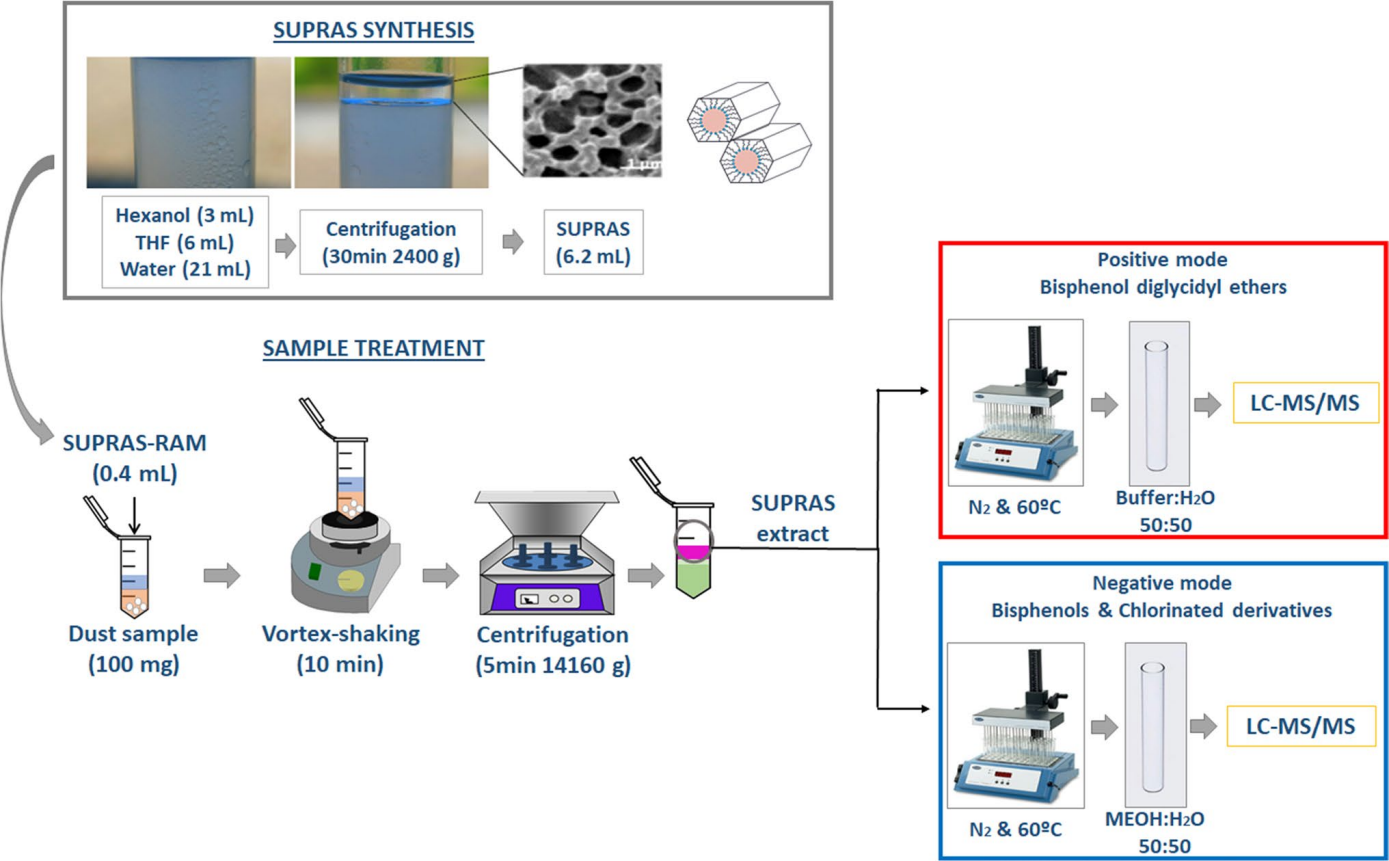
General scheme of the analytical methodology, the synthesis of the SUPRAS (upper part of the scheme) and sample treatment and quantification (at the bottom)

Dust samples were analysed according to the method previously described by Caballero-Casero et al. [21] with slight modifications. Briefly, 100 mg of dust sample was weighted in a 2-mL microtube Safe-Lock from Eppendorf Iberia (Madrid, Spain), and extracted with 0.4 mL of SUPRAS by vortex-shaking for 10 min at 2500 rpm. Three small glass balls (3 mm in diameter) were added to facilitate the extraction. Afterwards, the mixture was centrifuged at 14,160 g for 5 min (36 × 2.2/1.5 mL angle rotor high-speed brushless centrifuge MPW-350R from MPW Med- Instruments. Warschaw, Poland) to accelerate phase separation. Two aliquots of 0.075 mL of the SUPRASs extract were transferred to a 15-mL glass centrifuge tube. Both extracts were evaporated to dryness under a gentle nitrogen stream at 60 °C (~ 1 h) and the analytes were redissolved in 0.16 mL of methanol:water (50:50 v/v) or methanol:buffer (50:50 v/v) for bisphenols/chlorinated derivatives or diglycidyl ethers, respectively. Ammonium formate/formic acid (12.5 mM, pH 3.75) buffer was used to promote the formation of [M + NH_4_]^+^ diglycidyl ether adducts, which are necessary to improve diglycidyl ether sensitivity in MS/MS detection [22]. The extracts were introduced into a glass vial with an insert prior to being analysed by LC-ESI( ±)-MS/MS. Figure 2 shows an overall scheme of the analytical method.

### Quantification of bisphenols by LC–ESI–MS/MS

Bisphenols were quantified by liquid chromatography coupled to mass spectrometry in tandem (LC–MS/MS). For this purpose, a liquid chromatograph (Agilent HP 1200 series, Palo Alto, CA, USA) equipped with a binary solvent pumping system and an autosampler was used. Chromatographic separation was performed at 35 °C on a reverse-phase column ACE 3 C18-PFP, 150 mm × 3.0 mm, 3.5 µm (ACE, UK). It was preceded by a C18 Guard Cartridge ACE 3 C18-PFP, 3.0 mm × 4.6 mm, 4 μm (ACE, UK). The injection volume was 10 µL. Both bisphenols/halogenated derivatives and diglycidyl compounds were analysed under the same chromatographic conditions. The mobile phase consisted of water (A) and methanol (B) at a flow rate of 0.3 mL min^−1^, and the gradient elution was programmed as follows: starts at 50% of B and increases up to 60% in 2 min. Then, a linear gradient from 60 to 80% for 2 min and from 80 to 90% for 18 min, reaching 100% of B for 1 min. Finally, the column was equilibrated under the initial conditions for 5.5 min. Mass spectrometry analyses were accomplished by using a hybrid triple quadrupole/linear ion trap (Applied Biosystems MSD Sciex 4000QTRAP, Foster City, CA, USA) with a TurboIonSpray (TIS) interface. All data were acquired and processed using the Analyst 1.5.1 Software (Applied Biosystems). The MS/MS system was operated in multiple reaction monitoring (MRM) positive mode to quantify ammonium adducts of diglycidyl ethers. Bisphenols/chlorinated derivatives were determined by MRM negative ion mode. Quantitative analyses were carried out using two specific combinations of a precursor-product ion transition for each compound, with a dwell time set up at 30 ms. Common MS parameters were as follows: probe vertical y-axis position, 2 mm; probe horizontal y-axis position, 6 mm; curtain gas (N_2_), 27 psig; ion source gas 1 (nebulizer gas), 40 psig; ion source gas 2 (turbo gas), 55 psig; temperature of the turbo gas, 600ºC; ion spray voltage: ± 4500 V. Parameter values for the analyser were as follows: 1.0 unit resolution for the first and third quadrupoles; collision gas 3.0 × 10^−5^ Torr; collision energy − 26 V. The compound specific MS/MS parameters are shown in Table 1. Isotopic dilution calibration was performed for the quantification of bisphenol compounds, by analysing analytical standards in methanol:water (50:50, v/v) or methanol:buffer (50:50 v/v) for bisphenols/chlorinated derivatives or diglycidyl ethers, respectively. The internal standards _12_C^13^-BPA and d_6_-BADGE were respectively added.

### Analytical method performance

Calibration curves (*n* = 10) were built by analysing bisphenols and chlorinated derivative standard solution in methanol:water (50:50, v/v) and diglycidyl ethers in methanol:ammonium formate/formic acid buffer (12.5 mM, pH 3.75; 50:50 v/v) at a concentration range of 0.01–1000 ng mL^−1^. Signal variability was corrected with the signal of the internal standard _12_C^13^-BPA and d_6_-BADGE for bisphenols and diglycidyl ethers, respectively. The slopes of these calibration curves were compared with the slope of calibration curves prepared by standard addition, using an appropriate Student’s *t*-test [23].

The sensitivity of the method was calculated as three times of standard deviation of six blank determinations for the limit of detection (LOD) or ten times to calculate the limit of quantification (LOQ). The limits of the method were estimated from the respective LOD and LOQ taking into account the sample amount (100 mg), extraction conditions and recoveries obtained for each analyte.

Since a reference-certified material of bisphenols in dust is unavailable, method accuracy was evaluated by calculating the recoveries in six aliquots of pooled dust samples fortified with bisphenols, chlorinated derivatives and diglycidyl ethers and subjected to the whole analytical process. Precision of the method, calculated as intra-day variation, was calculated as the square root of the mean of the average variance value obtained for the six aliquots of pooled dust samples analysed, and expressed as relative standard deviation (RSD).

### Occupational exposure risk assessment to bisphenol compounds

In order to have a preliminary approach on the risk of exposure to bisphenols and derivatives of greenhouse workers, both the theoretical bioaccessibility (Ba) of the compounds and the average daily dose (ADD) via dust ingestion were estimated. Bioaccessibility can be defined as the fraction of the total amount of an ingested substance that becomes accessible for absorption through the epithelial layer of the gastrointestinal tract. The following equation, described by Dong et al. [24], allows the estimation of Ba when log *K*_ow_ value of bisphenols ranges between 5 and 8:

$$Ba=a+\frac{\left(b-a\right)*(8-logKow)}{8-5}$$

where the constants* a* and* b* are 0.2 and 0.8 respectively. For log *K*_ow_ > 5, Ba is assumed to be 0.8; whereas if log *K*_ow_ > 8, Ba is equal to 0.2.

In the greenhouse cultivation sector, farm labours are grouped in a season, based on the specific requirements of each crop type. For this reason, seasonal workers frequently move within the European Union (EU) or even other countries like Morocco for working the whole year. Thus, the duration of working hours and working days per year for seasonal workers is extremely difficult to estimate. In this study, the average daily dose of bisphenols has been calculated under the hypothesis of an average working week of 37 h for 270 working days per year in the EU and 40 years of work [25]. The ADD was calculated according to the following equation:

$$ADD=\frac{C*Ba*IngR*EF*ED*ET}{BW*AT}$$

where *C* is the concentration of bisphenol found in dust (ng g^−1^), *Ba* is the estimated theoretical bioaccessibility value for the compound; *IngR* is the ingestion of dust of an adult per day (0.05 g day^−1^) [26]; *EF* is the annual exposure frequency (270 days year^−1^); *ED* is the lifetime exposure duration (40 years); *ET* is the fraction of the day that seasonal workers spend working in greenhouses (0.38); *BW* is the average body weight of workers (70.8 kg [27]); and *AT* is the total number of days considered for ADD estimation (270 days year^−1^ multiplied for ED for non-carcinogenic effects) [28].

## Results and discussion

### Optimisation of bisphenol extraction from dust by hexanol-based SUPRAS-RAM

Hexanol-based SUPRAS-RAM is a nanostructured solvent that is spontaneously formed. The amphiphile molecules of hexanol self-assemble in THF above the critical aggregation concentration. The addition of water promotes their coacervation and SUPRAS separates from bulk solution in a lighter new phase [20]. This SUPRAS consists of inverted hexagonal aggregates where the hydroxyl groups of hexanol are surrounding the aqueous cavities and the hydrocarbon chains are dispersed in THF (Fig. 2). The synthetic environment of SUPRAS (i.e. THF/water ratio) determines the size of the water cavity of the aggregates, and consequently, both the chemical composition and properties of this SUPRAS are tuneable [19]. On the other hand, it behaves as a restricted access material (SUPRAS-RAM) by removing macromolecules from the extract through chemical and physical mechanisms. Thus, proteins precipitate by the action of THF (reduces the solution dielectric constant) and hexanol (forms mixed complex with proteins), while polar macromolecules are excluded by controlling the size of the aqueous cavity [29].

A pool of dust samples from samples 1–5 was prepared for the optimisation and in-house validation of the method. The main variables of the proposed analytical methodology affecting the quantification of bisphenols in dust were evaluated and optimised. The existing inter- and intra-variable relations are complex and unknown, so a multivariate analysis was performed. The variables were optimised based on the obtained results of the experiments programmed by the multivariant Box–Behnken response surface design model (Minitab Statistical Software. Free software. 20 version).

For the optimisation, 100mg of pooled dust sample and 10% of hexanol were established. The following variables were investigated: (i) the *percentage of THF* (10–60%) because it controls the size of the aqueous cavity, which has a significant impact on SUPRAS extraction and clean-up capabilities. The selection of the value range for THF was based on the hexanol-based SUPRAS phase diagram [20]. (ii) The *SUPRAS volume* (0.05–0.4 mL) to perform the extraction. The value range was established trying to reach a balance between maximum extraction efficiency and low limits of quantification for the method (MLQ). (iii) The *time of extraction* in a range of 1 to 30 min, the usual extraction times required in SUPRAS-methodologies. (iv) The *volume of the reconstitution solution for bisphenol solubilisation* (0.075–0.3 mL). The value range was selected under two considerations: to obtain enough volume for performing chromatographic analysis by triplicate (if necessary) and to achieve the highest possible concentration factors and recoveries for bisphenols.

Since the relations of the variables were unknown, all the responses from the different variables were considered equally important and the weight and importance were all set at 1. With these conditions, the Box–Behnken model proposed 27 experimental runs, including duplicates, with a random combination of the values of the studied variables. Values approaching 100% of total recovery (extraction+selectivity) were selected as optimal value, considering it as the target value. Table 2 shows the results obtained for the optimisation of extraction parameters, including fit values with their corresponding standard errors, the confidence and predicted intervals and desirability. The analysis of the obtained results gives optimal values for performing the extraction of bisphenols from dust 0.4mL of SUPRAS with a composition of 10% hexanol and 20% THF; and 0.16mL of reconstitution solution. Any influence related to the variable of extraction time was observed in the range of 5–30 min; thus, a time of 10 min was selected as optimal.

**Table 2:** Response optimisation obtained for bisphenols, chlorinated derivatives and diglycidyl ethers by multivariate analysis

Analyte	Fit	Standard error	Confidence interval (95%)	Predicted interval (95%)	Desirability
BPA	96	6.4	90.5–114.9	74.7–145.2	0.9638
BPB	97	18.9	46.5–98.2	33.2–184.9	0.9747
BPE	109	10.8	60.2–117.4	44.0–133.7	0.8313
BPF	99	7.7	66.3–130.4	59.8–186.9	0.9976
BPP	94	7.3	78.1–109.8	67.3–130.7	0.9440
BPS	95	9.53	61.1–119.6	76.8–146.9	0.9950
BPZ	93	11.0	70.8–118.7	54.4–135.1	0.9598
BPAF	106	7.7	86.0–119.5	74.6–131.4	0.9978
BPAP	107	9.9	74.0–117.5	59.1–132.4	0.9983
MCBPA	93	16.4	65.1–136.5	40.6–161.0	0.9476
DCBPA	87	10.3	60.2–95.1	44.8–120.5	0.9297
TCBPA	96	10.5	76.7–122.6	60.9–138.4	0.9585
TeCBPA	98	7.34	72.4–104.4	61.4–125.4	0.9687
BADGE	101	10.0	75.8–119.4	60.8–134.4	0.9676
BADGE·H2O	97	20.3	58.3–146.6	27.9–176.9	0.9489
BADGE·2H2O	107	13.9	66.7–117.3	52.8–132.3	0.9909
BADGE·HCl	90	7.3	74.1–105.7	63.2–116.6	0.9697
BADGE·2HCl	95	10.1	69.4–113.5	54.2–128.7	0.9140
BADGE·HCl·H2O	86	14.7	69.8–108.7	44.4–125.1	0.8448
BFDGE	102	9.5	80.4–121.9	66.2–136.1	0.9706
BFDGE·2H2O	108	9.9	78.2–121.6	63.2–136.6	0.9994

### Analytical method performance

The performance of the analytical method was evaluated according to the procedures specified in the section “Materials and methods”. The correlation coefficients (*r*) for the calibration curves were in the range 0.9808–0.9990, indicating a good fit (Table 3). The calculated Student´s *t*-values obtained for the comparison of the slopes of external calibration and the standard addition method (1.21–2.29) were all lower than the critical *t*-value (2.98) for a 95% confidence level. So, no significant differences were found between the calibration curves obtained by both methods, and consequently matrix components were not expected to interfere in the quantification of bisphenols in dust.

**Table 3:** Analytical parameters of the in-house method validation

Analyte	*t*_R_	*r*	LOD	LOQ	MQL	Recovery	RSD
	(min)		(ng mL^−1^)	(ng mL^−1^)	(ng g^−1^)	(%)	(%)
BPA	12.37	0.9945	0.015	0.04	0.04	96	4
BPB	12.92	0.9926	0.009	0.02	0.02	97	5
BPE	11.87	0.9980	0.011	0.04	0.03	109	5
BPF	11.28	0.9808	0.032	0.09	0. 08	99	2
BPP	16.69	0.9979	0.013	0.04	0.04	94	8
BPS	7.81	0.9990	0.009	0.03	0.03	95	7
BPZ	13.99	0.9990	0.010	0.03	0.03	93	1
BPAF	14.50	0.9988	0.011	0.04	0.03	106	1
BPAP	13.73	0.9931	0.012	0.04	0.03	107	3
MCBPA	13.40	0.9924	0.027	0.08	0.07	93	2
DCBPA	14.64	0.9962	0.017	0.05	0.05	87	15
TCBPA	15.77	0.9938	0.015	0.04	0.04	96	7
TeCBPA	17.01	0.9977	0.012	0.03	0.03	98	6
BADGE	15.72	0.9959	0.006	0.02	0.02	101	9
BADGE·H2O	12.65	0.9968	0.004	0.01	0.01	97	4
BADGE·2H2O	11.55	0.9925	0.033	0.09	0.07	107	6
BADGE·HCl	15.55	0.9978	0.051	0.13	0.12	90	6
BADGE·2HCl	15.17	0.9982	0.045	0.13	0.12	95	3
BADGE·HCl·H2O	13.22	0.9918	0.037	0.10	0.10	86	10
BFDGE	14.65	0.9948	0.051	0.15	0.13	102	4
BFDGE·2H2O	9.98	0.9892	0.046	0.11	0.09	108	7

*t*_*R*_ time of retention; *r* correlation coefficient; *LOD* instrumental limit of detection; *LOQ* instrumental limit of quantification; *MQL* method limit of quantification

The values obtained for LOD and LOQ ranged from 0.009 to 0.032 and 0.02 to 0.09 ng mL^−1^ for bisphenols, 0.012–0.027 and 0.03–0.08 ng mL^−1^ for chlorinated derivatives; and 0.004–0.051 and 0.01–0.15 ng mL^−1^ for diglycidyl ethers, respectively (Table 3). The MQL ranged from 0.02 to 0.08 and 0.03 to 0.07 ng g^−1^ for bisphenols and chlorinated derivatives; and 0.01–0.13 ng g^−1^ for diglycidyl ethers, respectively.

**Fig. 3:**
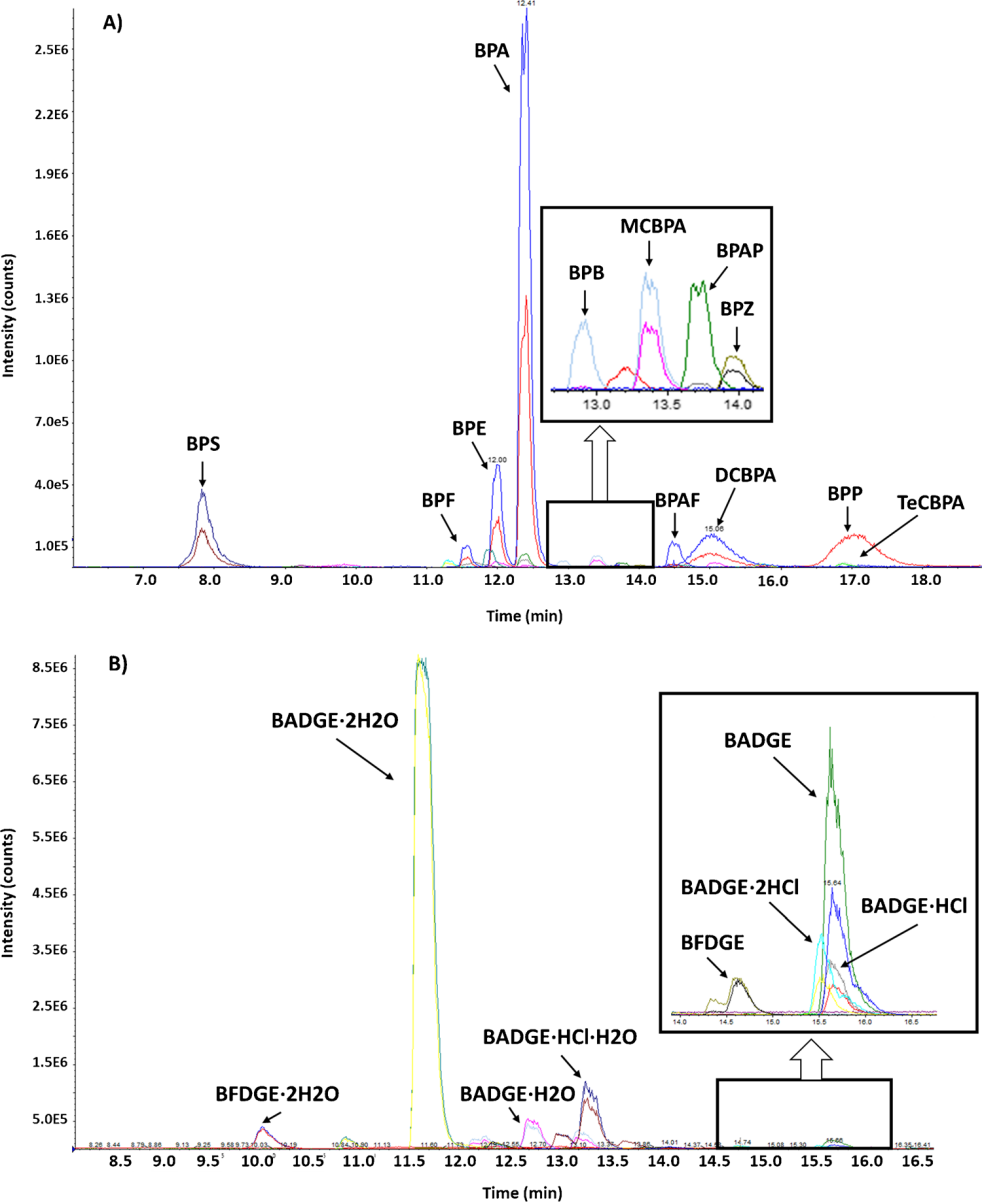
Typical LC–ESI–MS/MS chromatogram of a fortified dust sample. **a** Bisphenols and chlorinated bisphenols, and **b** diglycidyl ether compounds

Recoveries for six aliquots of pooled dust samples, fortified with bisphenols, chlorinated derivatives and diglycidyl ethers, were in the ranges 93–109 and 87–98% for bisphenols and chlorinated derivatives and 86–108% for diglycidyl ethers, respectively. A typical chromatogram of a dust sample fortified is shown in Fig. 3. Intra-day variability ranged between 1–8 and 2–15% for bisphenols and chlorinated derivatives and 3–10% for diglycidyl ethers, respectively. Table 3 shows the analytical parameter values for each bisphenol compound.

### Analysis of greenhouse dust samples

Five dust samples collected in two different greenhouses were analysed by the proposed analytical methodology. Table 4 shows the concentration found, expressed as the mean value of three determinations (ng g^−1^), along with their respective standard deviations. Only two bisphenols (BADGE·HCl and BADGE·2HCl) were not found in any of the analysed samples. As it was expected, BPA was the most abundant bisphenol in all the samples collected from the different sites and materials of the greenhouses (concentrations so high as 5275 ng g^−1^ were found), with 100% of frequency of detection. The second most abundant (up to 1850 ng g^−1^) was a derivative of BADGE (BADGE·H2O). Both high levels of concentration were detected in dust collected from lamps of the sawtooth greenhouse. A possible reason is the position of the lamps, which are hung from the ceiling in the centre of the greenhouse, which may make easier the deposition of contaminants coming from the whole greenhouse. On the other hand, it is interesting that the four bisphenol-chlorinated derivatives have been identified in all the samples. Plant protection and biocidal products may contribute to the BPA transformation in its chlorinated forms.

**Table 4:** Concentrations found of bisphenols, chlorinated and diglycidyl ethers along with their respective standard deviation and detection frequency in greenhouse dust

Analyte	Concentration found (ng g^−1^) ± SD					∑BPs^a^	DF (%)
	Sample 1	Sample 2	Sample 3	Sample 4	Sample 5		
BPA	5275 ± 33	223 ± 1	18 ± 2	125 ± 10	38.8 ± 0.1	5641	100
BPB	7.0 ± 0.5	2.2 ± 0.2	2.8 ± 0.2	1.48 ± 0.06	2.64 ± 0.07	13.48	100
BPE	< MQL	nd	nd	nd	3.2 ± 0.2	-	40
BPF	55 ± 1	nd	nd	6.6 ± 0.3	nd	61.60	40
BPP	4.9 ± 0.4	0.62 ± 0.05	4.1 ± 0.3	1.3 ± 0.1	37 ± 2	10.92	100
BPS	0.68 ± 0.07	nd	nd	nd	< MQL	0.68	40
BPZ	nd	nd	nd	nd	9.7 ± 0.3	-	20
BPAF	0.25 ± 0.01	0.22 ± 0.01	0.153 ± 0.003	0.336 ± 0.003	0.91 ± 0.02	0.96	100
BPAP	nd	< MQL	nd	< MQL	nd	-	40
MCBPA	38 ± 2	1.9 ± 0.1	< MQL	3.74 ± 0.09	0.30 ± 0.01	43.64	100
DCBPA	1.7 ± 0.2	0.41 ± 0.06	0.33 ± 0.06	0.67 ± 0.04	0.6 ± 0.1	3.11	100
TCBPA	0.81 ± 0.07	0.39 ± 0.03	0.291 ± 0.009	0.42 ± 0.03	0.51 ± 0.04	1.91	100
TeCBPA	0.46 ± 0.03	0.27 ± 0.02	0.25 ± 0.02	0.52 ± 0.03	0.28 ± 0.02	1.50	100
BADGE	nd	nd	nd	nd	0.60 ± 0.06	-	20
BADGE·H2O	148 ± 2	1.22 ± 0.07	0.69 ± 0.02	< MQL	0.84 ± 0.04	149.91	100
BADGE·2H2O	1850 ± 160	20 ± 1	12.4 ± 0.3	2.5 ± 0.2	9.5 ± 0.8	1882	100
BADGE·HCl	nd	nd	nd	nd	nd	-	0
BADGE·2HCl	nd	nd	nd	nd	nd	-	0
BADGE·HCl·H2O	305 ± 13	2.29 ± 0.03	4.1 ± 0.4	nd	4.3 ± 0.6	311.39	80
BFDGE	nd	nd	nd	nd	3.1 ± 0.3	-	20
BFDGE·2H2O	12 ± 1	5.2 ± 0.3	4.4 ± 0.2	1.2 ± 0.1	3.6 ± 0.3	22.8	100

^a^Sum of found concentration of bisphenol for sawtooth greenhouse (samples 1–4); *SD* standard deviation; *DF* detection frequency; *nd* non detected; *n* = 3

Regarding the type of greenhouse, unfortunately, there were no sufficient samples for the shade house type, so it was not possible to establish any correlation or comparison material/location/bisphenol concentration. However, focusing on samples collected from wall sample 2 and sample 5, of sawtooth and shade greenhouse, respectively; BPA concentration in sample 2 is five times higher than the detected in sample 5. Important parameters affecting chemical leaching from plastic materials to dust are different in both greenhouses, such as ventilation, humidity and radiation. Despite these different environmental conditions, the concentrations for the rest of the bisphenols were similar. Thus differences in BPA concentration could be more related to the material of the greenhouse.

The predominance of BPA and their derivatives in greenhouse dust on the rest of bisphenol analogues was clear (e.g. BPA, MCPA, BADGE·H_2_O, BADGE·2H_2_O, BADGE·HCl·H_2_O) although the concentrations of other bisphenols and their derivatives (e.g. BPF and BFDGE·2H_2_O) were significant. The obtained results bring to light the importance of evaluating the bisphenol risk exposure from greenhouses.

### *Greenhouse workers exposure to bisphenols *via* dust ingestion*

Humans are exposed to dust-related chemicals via inhalation, dermal and ingestion. Inhalation exposure to dust is minimum, thanks to the water spread spots located in the ceiling of greenhouses for humidity control, which reduces dust dispersion. On the other hand, due to the use of phytosanitary products, all workers are encouraged to wear individual protection equipment as part of the strict protocol of measures for the prevention of occupational risks. This, along with the lack of information related to the fraction of bisphenols that is absorbed by skin, the human exposure to bisphenols via dermal absorption was not considered in this study. Thus, we only focused on exposure via ingestion.

The estimated ADD values for each bisphenol compound are shown in Table 5. In the case of the sawtooth greenhouse, the ADD values have been calculated considering the sum of concentrations of found bisphenols from samples 1–4, while concentrations found in sample 5 were used for the estimation of ADD in the shade greenhouse. For the first greenhouse, BPA (47.81 ng kg^−1^ day^−1^) and BADGE·2H2O (15.95 ng kg^−1^ day^−1^) presented the highest average daily dose via dust ingestion, having both compounds a DF of 100%. However, in the shade greenhouse, the highest ADD were calculated for BPA (0.33 ng kg^−1^ day^−1^) and BPP (0.31 ng kg^−1^ day^−1^), while BADGE·2H2O (0.08 ng kg^−1^ day^−1^) was two orders of magnitude lower.

**Table 5:** Theorethical bioaccessibility values and the estimated average daily dose via dust ingestion for all the targeted bisphenols

Analyte	Ba	ADD sawtooth greenhouse (ng kg^−1^ day^−1^)	ADD shade greenhouse (ng kg^−1^ day^−1^)
BPA	0.8	47.81	0.33
BPB	0.8	0.11	0.02
BPE	0.8	-	0.027
BPF	0.8	0.52	-
BPP	0.49	0.09	0.31
BPS	0.8	0.006	-
BPZ	0.8	-	0.08
BPAF	0.8	0.008	0.008
BPAP	0.8	-	-
MCBPA	0.8	0.37	0.003
DCBPA	0.79	0.026	0.005
TCBPA	0.66	0.016	0.004
TeCBPA	0.52	0.013	0.002
BADGE	0.8	-	0.005
BADGE·H2O	0.8	1.27	0.007
BADGE·2H2O	0.8	15.95	0.08
BADGE·HCl	0.8	-	-
BADGE·2HCl	0.8	-	-
BADGE·HCl·H2O	0.8	2.64	0.03
BFDGE	0.8	-	0.026
BFDGE·2H2O	0.8	0.19	0.03

*Ba* theoretical bioaccessibility; *ADD* average daily dose

In 2018, the European Food Safety Authority (EFSA) reviewed the BPA exposure level considered as safe for humans and established a new tolerable daily intake (TDI) of 4·10^3^ ng kg^−1^ day^−1^ [30]. The estimated ADD for all bisphenols were below the suggested BPA TDI; thus, in theory, there is no risk chemical for greenhouse workers. However, it is necessary to consider that EFSA defines TDI as *the maximum amount of a substance to which any individual can be exposed every day of his/her life, through all possible sources, without any risk to his/her health* [30]. Moreover, previously reported ADD for indoor dust were much lower than the values estimated in this study. For example, Wang et al. [16] reported an average exposure to BPA via residential dust ingestion, calculated in samples collected from twelve countries, in the range 0.03–0.85 ng kg^−1^ day^−1^, which is up to four orders of magnitude lower than the estimated DDA for greenhouse dust (47.81–0.33 ng kg^−1^ day^−1^). This fact pointed out the great potential contribution of this source to the total exposure of greenhouse workers. However, due to the limited number of samples analysed in this study, the obtained results should warrant caution. More studies are required to assess the role that greenhouse dust plays in the bisphenol occupational exposure field.

## Conclusions

The exposure to bisphenol, chlorinated derivatives and diglycidyl ethers of humans is being strongly investigated by the Scientific Community. However, many potential sources of bisphenol exposure remain unknown. The analytical methodology proposed in this study, based on the use of SUPRAS and LC–MS/MS, has been successfully applied to the determination of twenty-one bisphenols in greenhouses dust. Building materials of greenhouses and the typical environmental conditions (high temperature, humidity, radiation, etc.) makes them potential sources of contaminants. Despite the calculated ADD values being under the tolerable daily intake proposed by EFSA, they were above the previously ADD values reported via indoor dust. Although it is difficult to draw definitive conclusions due to the reduced number of analysed dust samples, this study brings to light the importance of assessing the role that greenhouse dust plays in the bisphenol occupational exposure field for reducing the occupational risk exposure to a minimum.
